# Cytotoxic and Hypoglycemic Activity of Triterpenoid Saponins from *Camellia oleifera* Abel. Seed Pomace

**DOI:** 10.3390/molecules22101562

**Published:** 2017-09-21

**Authors:** Tai-Mei Di, Shao-Lan Yang, Feng-Yu Du, Lei Zhao, Tao Xia, Xin-Fu Zhang

**Affiliations:** 1 College of Horticulture, Qingdao Agricultural University, Qingdao 266109, China; dtmtea@163.com (T.-M.D.); shaolanyang@126.com (S.-L.Y.); zhaolei_tea@163.com (L.Z.); 2 College of Chemistry and Pharmacy, Qingdao Agricultural University, Qingdao 266109, China; fooddfy@126.com; 3 State Key Laboratory of Tea Plant Biology and Utilization, Anhui Agricultural University, Hefei 230036, China; xiatao62@126.com

**Keywords:** *Camellia oleifera*, triterpenoid saponin, oleiferasaponin A_3_, cytotoxic activity, hypoglycemic activity

## Abstract

One new and three known triterpenoid saponins were isolated and identified from *Camellia oleifera* seeds through IR, NMR, HR-ESI-MS and GC-MS spectroscopic methods, namely oleiferasaponin A_3_, oleiferasaponin A_1_, camelliasaponin B_1_, and camelliasaponin B_2_. The structure of oleiferasaponin A_3_ was elucidated as 16α-hydroxy-21β-*O*-angeloyl-22α-*O*-cinnamoyl-23α-aldehyde-28-dihydroxymethylene-olean-12-ene-3β-*O*-[β-d-galactopyranosyl-(1→2)]-[β-d-xylopyranosyl-(1→2)-β-d-galactopyranosyl-(1→3)]-β-d-gluco-pyranosiduronic acid. Camelliasaponin B_1_ and camelliasaponin B_2_ exhibited potent cytotoxic activity on three human tumour cell lines (human lung tumour cells (A549), human liver tumour cells (HepG2), cervical tumour cells (Hela)). The hypoglycemic activity of oleiferasaponin A_1_ was testified by protecting pancreatic β-cell lines from high-glucose damage.

## 1. Introduction

Triterpenoid saponins are vital plant secondary metabolites that have been applied to cosmetics [1,2], agriculture [3], and medicine [4,5] for their diverse biological and pharmacological activities. *Camellia oleifera* was named for its seeds with plentiful edible oil. Tea seed pomace—the byproduct of oil manufacture—contains about 8% saponins, which have historically been wasted without full use [6]. In recent years, some research works concerning the extraction, structures, and activity identification of saponins obtained from *Camellia oleifera* seed have been published. There are 11 novel triterpenoid saponin compounds obtained from *Camellia oleifera* seed [6,7,8,9,10,11,12,13]. Meanwhile, their cell protective activity [14], antioxidant activity [10,15], anti-fungal activity [16,17], cytotoxic activity [11,12,13,18] have been reported, indicating that the different activities depend on the different compound structures. Approximately 30 types of saponin were indicated by liquid chromatography–mass spectrometry (LC-MS) analysis in the seed pomace of *Camellia oleifera* [17]. Therefore, it is significant to continue extracting, identifying, and exploring the biological and pharmacological activities of saponins in *Camellia oleifera* seed pomace.

One new triterpenoid saponin (oleiferasaponin A_3_) and three known saponins (oleiferasaponin A_1_, camelliasaponin B_1_, and camelliasaponin B_2_) were isolated from the tea seed pomace of *Camellia oleifera* in our study. We observed that camelliasaponin B_1_ and camelliasaponin B_2_ significantly inhibited the proliferation of human lung cancer cells (A549), human liver cancer cells (HepG2), cervical cancer cells (Hela), especially A549 cell lines. In addition, a glucose-stimulated insulin secretion (GSIS) experiment indicated that oleiferasaponin A_1_ possessed protective activity on pancreatic β-cell lines injured by high-glucose. Our results will promote further application of oleanane-type saponins in pharmacology.

## 2. Results

### 2.1. The Characterization of the Oleiferasaponin A_3_

**Figure 1:**
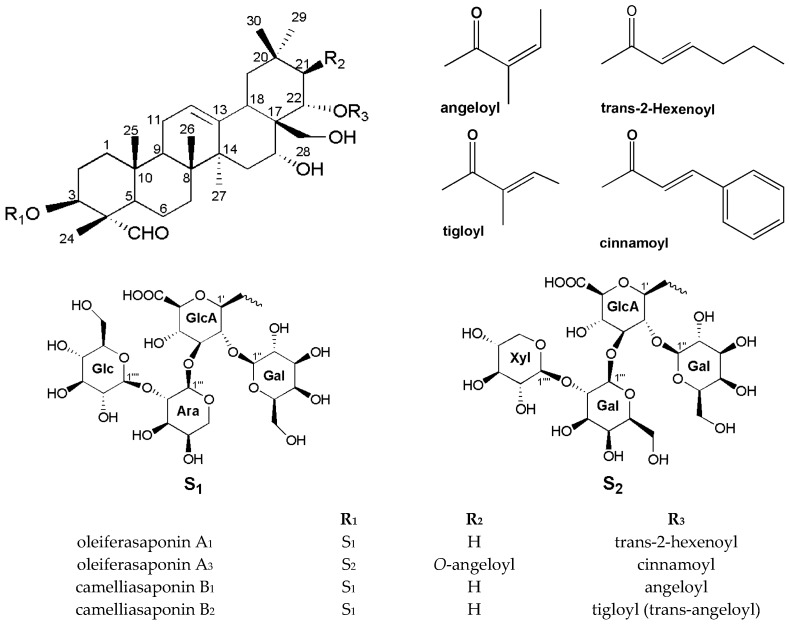
Structure of compounds.

One new and three known oleanane-type saponins were obtained, the structures of which were further deduced mainly by the data of IR, NMR, HR-ESI-MS and GC-MS experiments (Figure 1), the spectrums can be found in supplementary materials.

**Figure 2:**
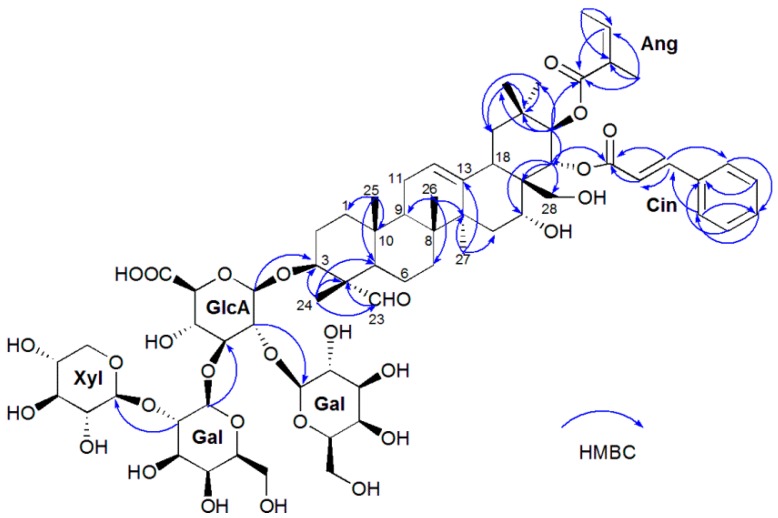
Key HMBC correlations of oleiferasaponin A_3_.

The molecular formula C_67_H_96_O_28_ of oleiferasaponin A_3_ was determined from the HR-ESI-MS [M − H]^−^ ion peak at *m*/*z* 1347.5980. The IR spectrum of oleiferasaponin A_3_ showed absorption bands at 3371 and 1618 cm^−1^, ascribable to hydroxyl and olefinic groups, and broad bands at 1042 cm^−1^, suggestive of an ether functional group. The NMR (Table 1) data of oleiferasaponin A_3_, along with the HSQC spectrum, showed the correlation between the anomeric proton signals of sugar units at *δ*_H_, 4.38 (1H, d, *J* = 7.7 Hz, H-1′), 4.53 (1H, d, *J* = 6.1 Hz, H-1′′′′), 5.05 (1H, d, *J* = 7.9 Hz, H-1′′), 5.07 (1H, d, *J* = 7.7 Hz, H-1′′′), and *δ*c 103.4 (C-1’ of glucuronopyranosyl, GlcA), 106.2 (C-1’’’’ of xylopyranosyl, Xyl), 101.2 (C-1′′ of galactopyranosyl, Gal), 100.2 (C-1′′′ of Gal), respectively, which indicated the presence of four sugar residues. In addition, a cinnamoyl group (*δ*_H_ 6.49, 7.72 (1H, d, *J* = 16.0 Hz, Cin-H-2, Cin-H-3), 7.42, 7.42, 7.42, 7.60, 7.60 (1H, m, Cin-H-6, 7, 8, 9, 5), 117.4 (Cin-C-2), 127.8 (Cin-C-5, 9), 128.6 (Cin-C-6, 8), 130.1 (Cin-C-7), 134.4 (Cin-C-4), 145.3 (Cin-C-3), and *δ*_C_ 167.6 (Cin-C-1)), and an angeloyl group (*δ*_H_ 6.02 (1H, q, 22-O-Ang-3), 1.85 (3H, d, *J* = 7.2 Hz, 22-O-Ang-4), 1.82 (3H, s, 22-O-Ang-5)), which are also present in isotheasaponins B_3_ isolated from the leaves of the tea plant *Camellia sinensis* var*. sinensis* [19]. The remaining ^1^H and ^13^C-NMR signals, corresponding to a triterpene aglycon, showed the presence of six methyls (*δ*_H_ 0.93, 0.97, 1.05, 1.14,1.19, and 1.53 (3H, each, all s, H3-29, 26, 25, 30, 24, 27)), eight methylenes (*δ*_H_ 0.95, 1.55, 1.15, 1.73, 1.25, 2.68, 1.29, 1.68, 1.39, 1.72, 1.83, 2.09, 1.95, and 2.00 (2H, both m, H2-6, 1, 19, 7, 15, 2, 11), 3.02, 3.33 (2H, d, *J* = 10.8 Hz, H2-28)), including an oxygenated one, seven sp^3^ methines (four are oxygenated) (*δ*_H_ 1.38, 1.82, 2.71, and 3.90 (1H, each, all m, H1-5, 9, 18, 3), 5.62, 6.04 (1H, d, *J* = 10.2 Hz, H1-22, 21), 4.06 brs of H1-16), six sp^3^ quaternary carbon (*δ*_C_ 35.4, 35.6, 39.9, 41.1, 47.2, and 55.0 (C-20, 10, 8, 14, 17, 4), one tri-substituted double bond at *δ*_C_ 141.6 of C-13), and an aldehyde carbonyl (*δ*_H_ 9.50 (1H, s, H-23)). The positions of the Ang, Cin group and sugar components in oleiferasaponin A_3_ were clarified by an HMBC experiment (Figure 2), which showed a correlation between *δ*_H_ 6.04 (10.2) (H, d, H-21) and *δ*_C_ 168.1 of Ang-C-1, *δ*_H_ 5.62 (10.2) (H, d, H-22) and *δ*_C_ 167.6 of Cin-C-1, as well as GlcA-H-1′ and *δ*_C_ 84.7 (C-3 of the aglycone), Gal-H-1′′ and *δ*_C_ 77 (GlcA-C-2′), Gal-H-1′′′ and *δ*_C_ 81.6 (GlcA-C-3′), Xyl-H-1′′′′ and *δ*_C_ 82.4 (Gal-C-2’). 

The NOESY spectrum showed the cross peaks between H-22 at *δ*_H_ 5.62 and H-30 at *δ*_H_ 1.14, as well as those between H-16 at *δ*_H_ 4.06 and H-28 at *δ*_H_ 3.02, 3.33, suggesting that H-22 and H-16 are both β-oriented; that is, Cin group at C-22 and 16-OH group are both α-orientations. The H-3 at *δ*_H_ 3.90 correlated with H-23 at *δ*_H_ 9.50 and H-21 at *δ*_H_ 6.04 correlated with H-29 at *δ*_H_ 0.93, indicating that the glycosidic chain group at C-3 and Ang group at C-21 are β-configured. The absolute configuration of sugars of oleiferasaponin A_3_ was confirmed by acid hydrolysis and GC-MS analysis, which revealed one unit of D-glucuronic acid (GlcA), two units of D-galactose (Gal) and one unit of D-xylose (Xyl) [13,20]. Synthesizing the above analysis of all the proton and carbon signals, we established the structure of oleiferasaponin A_3_ as 16α-hydroxy-21β-*O*-angeloyl-22α-*O*-cinnamoyl-23α-aldehyde-28-dihydroxymethylene-olean-12-ene-3β-*O*-[β-d-galactopyranosyl-(1→2)]-[β-d-xylopyranosyl-(1→2)-β-d-galactopyranosyl-(1→3)]-β-d-gluco-pyranosiduronic acid. The other three known compounds were oleiferasaponin A_1_ (22-*O*-*cis*-2-hexenoyl-A_1_-barrigenol 3-*O*-[β-d-galactopyranosyl (1→2)][β-d-glucopyranosyl (1→2)-α-l-arabinopyranosyl (1→3)]-β*-*d-gluco-pyranosiduronic acid) [10], camelliasaponin B_1_ (22-*O*-angeloyl-A_1_-barrigenol 3-*O*-[β-d-galactopyranosyl (1→2)][β-d-glucopyranosyl (1→2)-α-l-arabinopyranosyl (1→3)]-β-d-gluco-pyranosiduronic acid) and camelliasaponin B_2_ (22-*O*-trans-angeloyl-A_1_-barrigenol 3-*O*-[β-d-galactopyranosyl (1→2)][β-d-glucopyranosyl (1→2)-α-l-arabinopyranosyl (1→3)]-β-d-gluco-pyranosiduronic acid) [20].

### 2.2. Anti-Proliferative Activity

**Figure 3:**
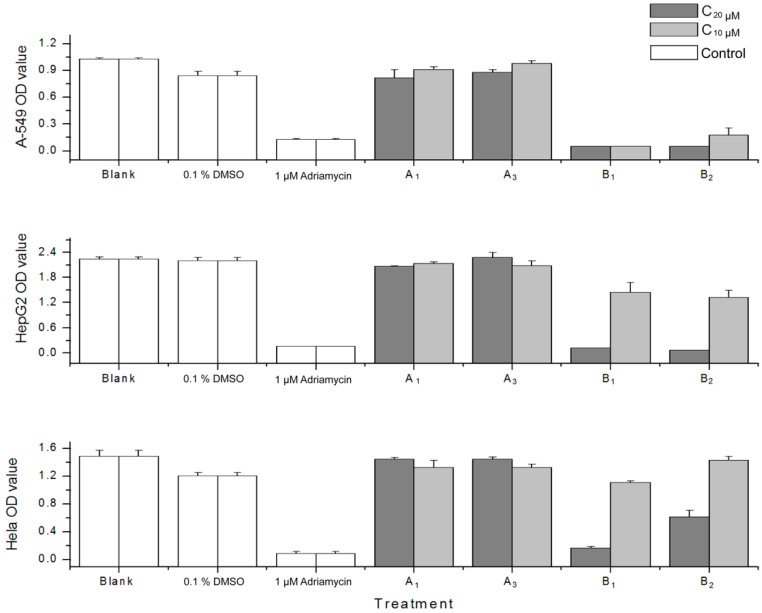
The effect of compounds on tumour cells proliferation. A_1_: Oleiferasaponin A_1_; A_3_: Oleiferasaponin A_3_; B_1_: Camelliasaponin B_1_; B_2_: Camelliasaponin B_2_.

Oleiferasaponin A_1_, oleiferasaponin A_3_, camelliasaponin B_1_ and camelliasaponin B_2_ obtained from *Camellia oleifera* seed pomace were tested against three human tumour cell lines (A549, Hela, HepG2) using cell proliferation bioassay (SRB). Camelliasaponin B_1_ and camelliasaponin B_2_ at the concentration of 20 μM exhibited effective anti-proliferative activity on the human tumour cell lines tested (Figure 3)—the inhibition ratios were more than 50%. Camelliasaponin B_1_ and camelliasaponin B_2_ at the concentration of 10 μM significantly inhibited the proliferation of human lung cancer cells (A549) (Figure 3)—the inhibition ratios were 94.44% and 79.12%, respectively. Our results indicated that camelliasaponin B_1_ and camelliasaponin B_2_ possessed potent cytotoxic activity. There are some reports about the structure–activity relationships of triterpenoid saponins [21,22].

The structures of camelliasaponin B_1_ and camelliasaponin B_2_ are similar, except for the orientation of C-22 angeloyl. Compared to previously reported results [11,12,13], it seems that the main groups contributing cytotoxicity are the C-22 Ang group and the C-28 free hydroxy group. As a result, the cytotoxic activity is a combined effect of sugar moieties and aglycone, rather than an isolated structural effect. Oleiferasaponin A_1_ and oleiferasaponin A_3_ did not show cytotoxic activity.

### 2.3. Hypoglycemic Activity

**Figure 4:**
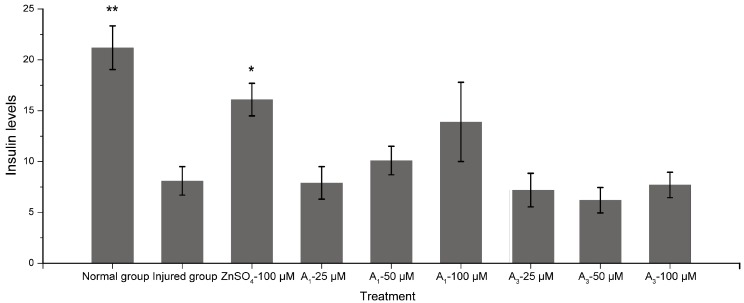
Cell protective effects of oleiferasaponin A_1_ and oleiferasaponin A_3_ on RIN-m5f cells injured by high-glucose. The values are expressed as mean ± SD. * *p* < 0.05, ** *p* < 0.01 with respect to the injured group.

Diabetes mellitus (DM) is the third most prevalent disease globally, and manifests as a disorder of blood glucose caused by metabolic disorder, which can induce cardiovascular system diseases and cancer, then threatening human health and life. Many studies regarding the cytotoxic activity of triterpenoid saponins have been reported [11,12,13], while few have been conducted concerning hypoglycemic activity [23]. Oleiferasaponin A_1_ and oleiferasaponin A_3_ did not exhibit cytotoxic activity on three human tumour cell lines (A549, Hela, HepG2), so we carried out a hypoglycemic activity study for further exploration of structure–activity relationship. Oleiferasaponin A_1_ and oleiferasaponin A_3_ were tested for their protective effect on RIN-m5f (islet-β cells) injured by high glucose. The insulin content of RIN-m5f cells upon treatment under 16.7 mmol/L glucose are shown below (Figure 4). With higher oleiferasaponin A_1_ concentration, the insulin levels of RIN-m5f (islet-β cells) was enhanced, which indicates that oleiferasaponin A_1_ has potential hypoglycemic activity against the damage induced by high glucose, and oleiferasaponin A_1_ may be a therapeutic agent for hyperglycemia treatment. Regarding the oleiferasaponin A_3_ group, no improvement effect on insulin levels was found in RIN-m5f (islet-β cells) injured by high glucose, even at a concentration of 100 μM. The difference of bioactivity between oleiferasaponin A_1_ and oleiferasaponin A_3_ is due to the different structure, including aglycone and sugar moieties (Figure 1). Compared with oleiferasaponin A_3_, we infer that the trans-2-hexenoyl group of oleiferasaponin A_1_ at C-22 may influence the activities, cooperating with sugar moieties.

## 3. Materials and Methods 

### 3.1. General

HPLC was run on Agilent 1260 HPLC (Agilent, Palo Alto, CA, USA). IR (infrared) spectra was recorded on Nicolet iN10 (Thermo Scientific Instrument Co., Boston, MA, USA) with KBr pellets. NMR spectra was measured on an AVANCE III (600 MHz) spectrometer (Bruker, Fallanden, Switzerland) using methanol-d_4_ (Sigma-Aldrich St. Louis, MO, USA) as solvent. HR-ESI-MS were determined on an electrostatic field orbital trap mass spectrometer (Thermo Scientific, Bremen, Germany) using an ESI source.

### 3.2. Plant Material

Tea seed pomace (*Camellia oleifera*) was collected from a factory in Shucheng, Anhui province, China. The plant material was identified by one of the authors (Associate Prof. X.F. Zhang), and was deposited in State Key Laboratory of Tea Plant Biology and Utilization, Anhui Agricultural University.

### 3.3. Extraction and Isolation

The samples were extracted and isolated according to Zhang et al. [10]. The tea seed powder 10 kg) was extracted three times with methanol at 60 °C under reflux each for 3 h. Concentrated solution (1.3 kg) was obtained after solvent evaporation under reduced pressure. The methanol extract (1.0 kg) was suspended in water and purified by nanofiltration membrane (SJM, Hefei, Anhui, China). Then, the purified solution (0.6 kg) was successively subjected to AB-8 macroporous resin column (Bonc, Cangzhou, Herbei, China), ordinary-phase silica gel column to yield a high-purity fraction (0.96 g), which was purified by HPLC (MeOH:H_2_O, 30:70) to furnish two saponin mixtures (Fr. 1, 0.13 g; Fr. 2, 0.17 g). The first faction was further purified by HPLC (acetonitrile-0.2% AcOH:H_2_O, 41:59, *v/v*) to afford oleiferasaponin A_1_ (8.7 mg) and oleiferasaponin A_3_ (3.9 mg). The second faction was further purified by HPLC (acetonitrile-0.2% AcOH:H_2_O, 37:63, *v/v*) to camelliasaponin B_1_ (11.6 mg) and camelliasaponin B_2_ (9.9 mg).

### 3.4. Acid Hydrolysis and GC-MS Analysis

Oleiferasaponin A_3_ was dissolved in 1 M HCI (Guoyao chemical reagent Co. Ltd, Beijing, China) (1 mL) for 3 h at 90 °C, then extracted with chloroform (Guoyao chemical reagent Co. Ltd, Beijing, China). The aqueous phase was evaporated under N_2_ flow. The residue was dissolved in 0.2 mL pyridine (Aladdin Industrial Co. Shanghai, China) containing L-cysteine methyl ester hydrochloride (10 mg/mL) and reacted at 70 °C for 1 h, then evaporated under N_2_ flow again. After concentrated, 0.2 mL trimethylsilylimidazole (Aladdin Industrial Co. Shanghai, China) was added for derivatization reaction, and reacted at 70 °C for another 1 h. The reaction mixture was partitioned between n-hexane and water. The organic phase was analysed by GC-MS (Agilent, Palo Alto, CA, USA) (injector temperature at 280 °C; the initial oven temperature was 160 °C for 1 min, linearly increased to 200 °C at 6 °C/min, then a further linear increase to 280 °C at 3 °C/min and held for 5 min). The standard sugar samples were subjected to the same reaction and GC-MS conditions.

### 3.5. Cytotoxic Activity Assay

#### 3.5.1. Cell Culture 

Human lung tumour cell (A549) lines, human liver tumour cell (HepG2) lines, and cervical tumour cell (Hela) lines were obtained from Qingdao Marine Biomedical Research Institute Limited by Share Ltd Testing Center (Qingdao, Shandong, China). Cells were cultured in DMEM complete medium supplemented with 10 % fetal bovine serum, 2 mM l-glutamine, 100 U·mL^−1^ penicillin, and 100 μg·mL^−1^ streptomycin at 37 °C in a 5% CO_2_ humidified atmosphere. The culture medium was refreshed every other day. After 80% of the cells were fused, cells were kept in logarithmic phase by trypsinization and subculturing.

#### 3.5.2. Cell Viability Assay

Human tumour cell lines in logarithmic phase were seeded in a 96-well plate at 4 × 10^3^ cells per well (180 μL per well), and incubated for 24 h. After 24 h, negative control without additions; solvent control was supplied with 0.1% DMSO; positive control with 1 μM adriamycin; 20 μM, 10 μM saponins were added to trial group, all incubated for 72 h. Then, 50% (*m/v*) ice-cold trichloroacetic acid was added to the medium for fixed cells. After staining by sulforhodamine B, tris solution (150 μL per well) was added to culture medium. Absorbance values were measured at 540 nm using an enzyme-linked immunosorbent reader (SpectraMax i3, Molecular Devices, San Francisco, CA, USA). The inhibition rate of cell proliferation was calculated as:

Inhibition rate (%) = [(OD_540_ (control group) − OD_540_ (trial group)) / OD_540_ (control group)] × 100% (1)

### 3.6. Hypoglycemic Activity Assay

Pancreatic β-cell lines (RIN-m5f) were obtained from Qingdao Marine Biomedical Research Institute Limited by Share Ltd. Testing Center (Qingdao, Shandong, China). Cells were cultured in RPMI-1640 complete medium supplemented with 10% fetal bovine serum, 1% penicillin–streptomycin and 1% glutamine. Then, pancreatic β-cell lines (RIN-m5f) were seeded in a 96-well plate (1 × 10^4^ cells per well). Cells were set in four groups: normal group with 5.5 mmol L^−1^ glucose; injured group with 16.7 mmol L^−1^ glucose; trial group with 16.7 mmol L^−1^ glucose and different concentrations (25, 50, 100 μM) of oleiferasaponin A_1_; positive control group with 16.7 mmol/L glucose and 100 μM ZnSO_4_. Each group was set three parallels and incubated for 48 h. Next, the medium was removed, cleaning twice with polybutylene succinate (PBS). The cells were incubated in medium with 5.5 mmol L^−1^ glucose for 1 h. Then, the medium was replaced by medium with 33.3 mmol L^−1^ glucose and incubated for 2 h. The supernatant was collected for insulin content detection using ELISA kit (CEA448Ra, Cloud-Clone Corp, Houston, TX, USA).

## 4. Conclusions

Four triterpenoid saponins were isolated from *Camellia oleifera* Abel. seed pomace: oleiferasaponin A_3_, oleiferasaponin A_1_, camelliasaponin B_1_, and camelliasaponin B_2_. The structure of oleiferasaponin A_3_ was identified. Camelliasaponin B_1_ and camelliasaponin B_2_ exhibited potent cytotoxic activity on three human tumour cell lines (A549, HepG2, Hela). Oleiferasaponin A_1_ possessed potential hypoglycemic activity protecting pancreatic β-cell lines from high-glucose damage.

## Figures and Tables

**Table 1:** NMR spectroscopic data for oleiferasaponin A_3_ (in methanol-d_4_).

Position	*δ*_C_	*δ*_H_	Position	*δ*_C_	*δ*_H_
1	38	1.15 m, 1.73 m	21-*O*-Ang		
2	24.3	1.83 m, 2.09 m	Ang-1	168.1	
3	84.7	3.90 m	Ang-2	128	
4	55		Ang-3	137.2	6.02, q (7.2)
5	47.4	1.38 m	Ang-4	14.6	1.85, d (7.2)
6	19.8	0.95 m, 1.55 m	Ang-5	19.5	1.82
7	31.8	1.29 m, 1.68 m	22-*O*-Cin		
8	39.9		Cin-1	167.6	
9	46.6	1.82 m	Cin-2	117.4	6.49 d (16.2)
10	35.6		Cin-3	145.3	7.72 d (16.2)
11	23.2	1.95 m, 2.00m	Cin-4	134.4	
12	123.6	5.44 m	Cin-5, 9	127.8	7.60 m
13	141.6		Cin-6, 8	128.6	7.42 overlap
14	41.1		Cin-7	130.1	7.42 overlap
15	33.4	1.39 m, 1.72 m	GlcA-1′	103.4	4.38 (7.8)
16	68.1	4.06 brs	GlcA-2’	77	3.79 overlap
17	47.2		GlcA-3’	81.6	3.90 overlap
18	39.4	2.71 m	GlcA-4’	69.6	3.56 overlap
19	46.4	1.25 m, 2.68 m	GlcA-5’	75.6	3.64 overlap
20	35.4		GlcA-6’	174.9	
21	78.4	6.04 d (10.2)	Gal-1′′	101.2	5.05 d (7.8)
22	73.8	5.62 d (10.2)	Gal-2′′	73.6	3.51 m
23	209.3	9.50 s	Gal-3′′	75.1	3.82overlap
24	9.4	1.19 s	Gal-4′′	69.6	3.84 overlap
25	15	1.05 s	Gal-5′′	76.4	3.33 m
26	15.9	0.97 s	Gal-6′′	63	3.02 d (10.8), 3.30 m
27	26.3	1.53 s	Gal-1′′′	100.2	5.07 d (7.8)
28	63.1	3.02 d (10.8), 3.33 d (10.8)	Gal-2′′′	82.4	3.68 overlap
29	28.2	0.93 s	Gal-3′′′	75.5	3.57 overlap
30	18.8	1.14 s	Gal-4′′′	69.1	3.83 m
			Gal-5′′′	76.4	3.65 overlap
			Gal-6′′′	61.2	3.71 overlap, 3.81overlap
			Xyl-1′′′′	106.2	4.53 d (7.8)
			Xyl-2′′′′	74.9	3.31 m
			Xyl-3′′′′	76.9	3.90 m
			Xyl-4′′′′	70.3	3.55 m
			Xyl-5′′′′	65.9	3.99 m, 3.21 m

^1^H (*δ* ppm, *J* in Hz, s: Single peak; d: Double peaks; m: Multipeaks) and ^13^C-NMR (*δ* ppm).

## Supplementary Materials

HR-ESR-MS and NMR spectra data of oleiferasaponin A_3_ can be accessed online.
