# Supplementary material for: Cytotoxic and Hypoglycemic Activity of Triterpenoid Saponins from Camellia oleifera Abel. Seed Pomace
Source: Molecules. 2017 Sep 21;22(10):1562. doi: 10.3390/molecules22101562 (PMC6151584; doi:10.3390/molecules22101562)

# SUPPLEMENTAL MATERIAL

**Supplemental Figure S1:** IR spectrum of oleiferasaponin $A_3$

**Supplemental Figure S2:** Mass spectra of oleiferasaponin $A_3$

**Supplemental Figure S3:** $^1$H-NMR spectrum of oleiferasaponin $A_3$

**Supplemental Figure S4:** $^{13}$C-NMR spectrum of oleiferasaponin $A_3$

**Supplemental Figure S5:** COSY spectrum of oleiferasaponin $A_3$

**Supplemental Figure S6:** HSQC spectrum of oleiferasaponin $A_3$

**Supplemental Figure S7:** HMBC spectrum of oleiferasaponin $A_3$

**Supplemental Figure S1:** IR spectrum of oleiferasaponin $A_3$

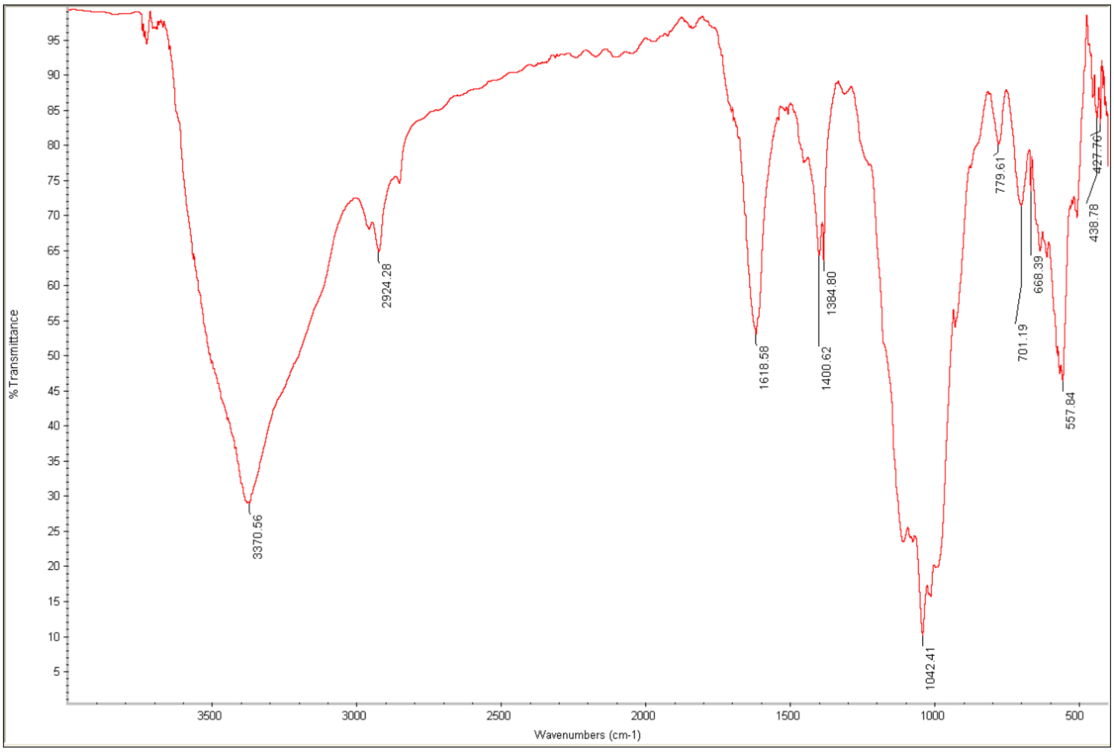

**Supplemental Figure S2:** Mass spectra of oleiferasaponin $A_3$

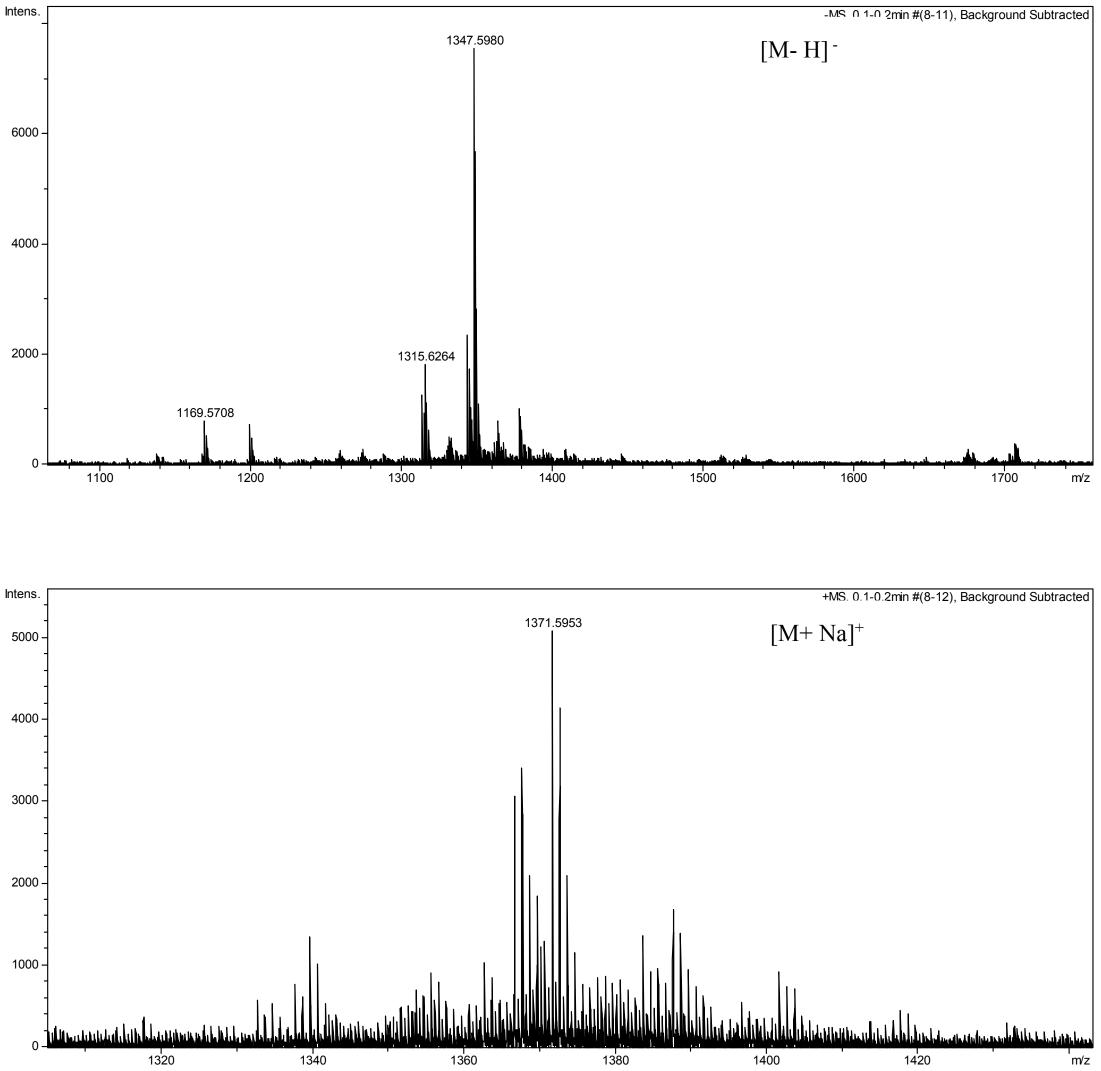

**Supplemental Figure S3:** $^{1}$H-NMR spectrum of oleiferasaponin $A_3$

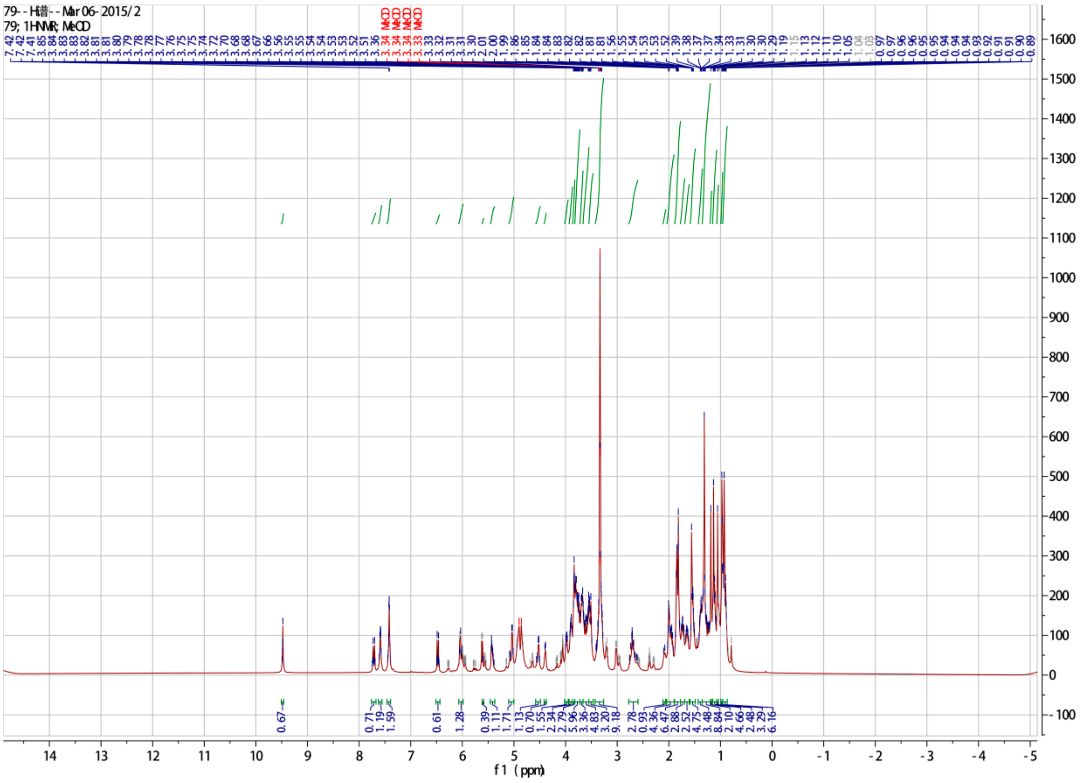

**Supplemental Figure S4:** $^{13}$C-NMR spectrum of oleiferasaponin $A_3$

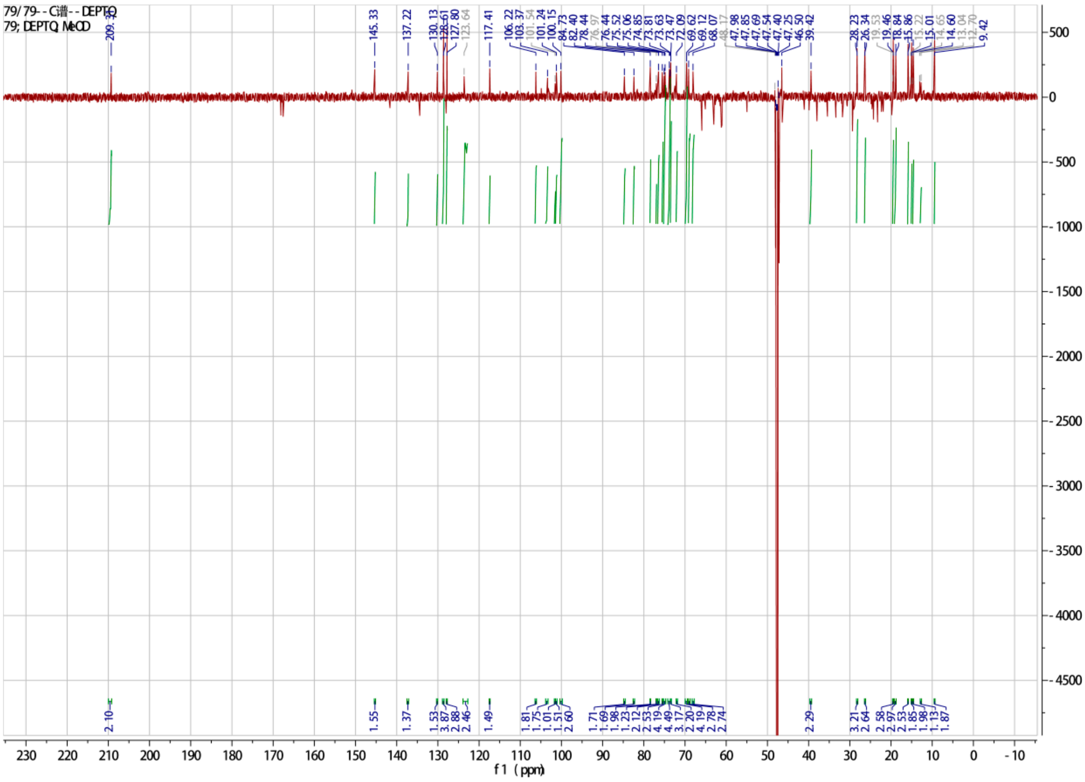

**Supplemental Figure S5:** COSY spectrum of oleiferasaponin $A_3$

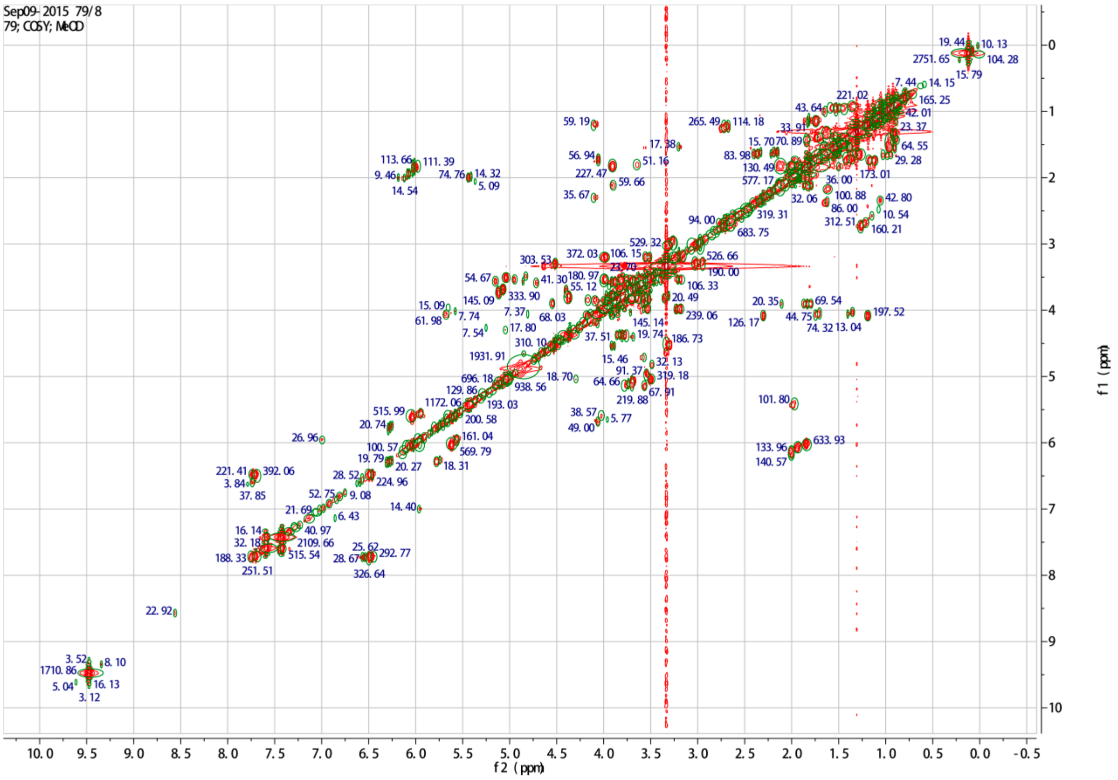


**Supplemental Figure S6:** HSQC spectrum of oleiferasaponin $A_3$

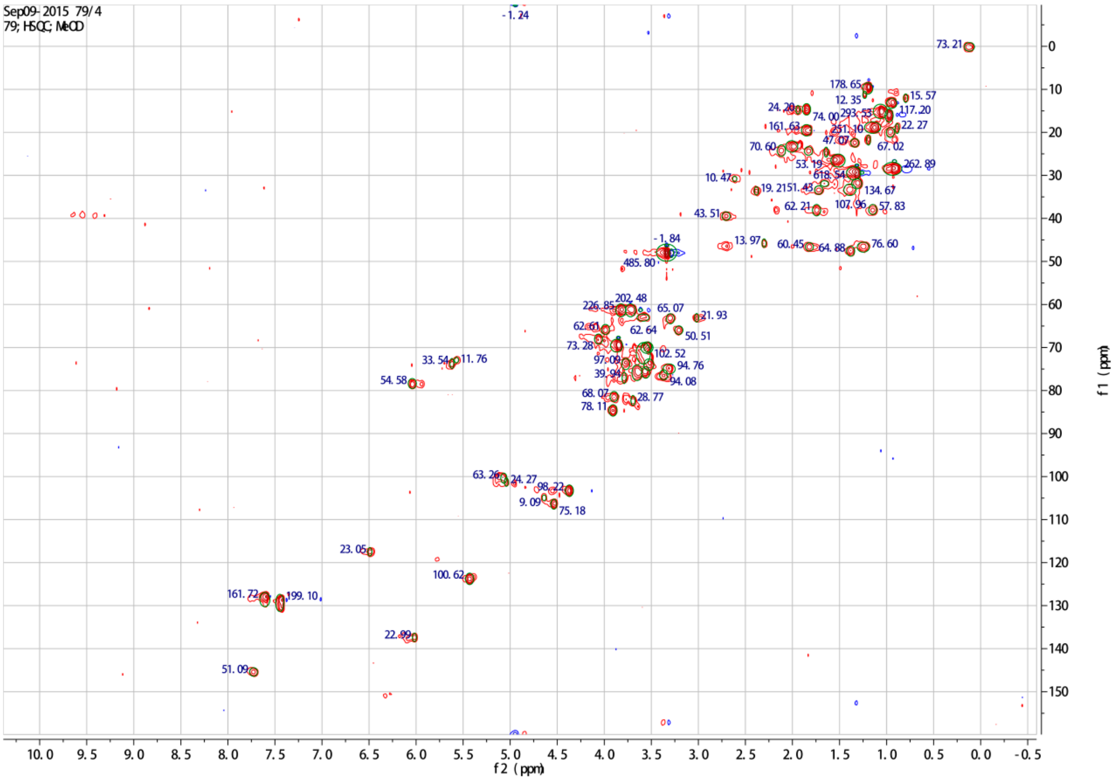

**Supplemental Figure S7:** HMBC spectrum of oleiferasaponin $A_3$

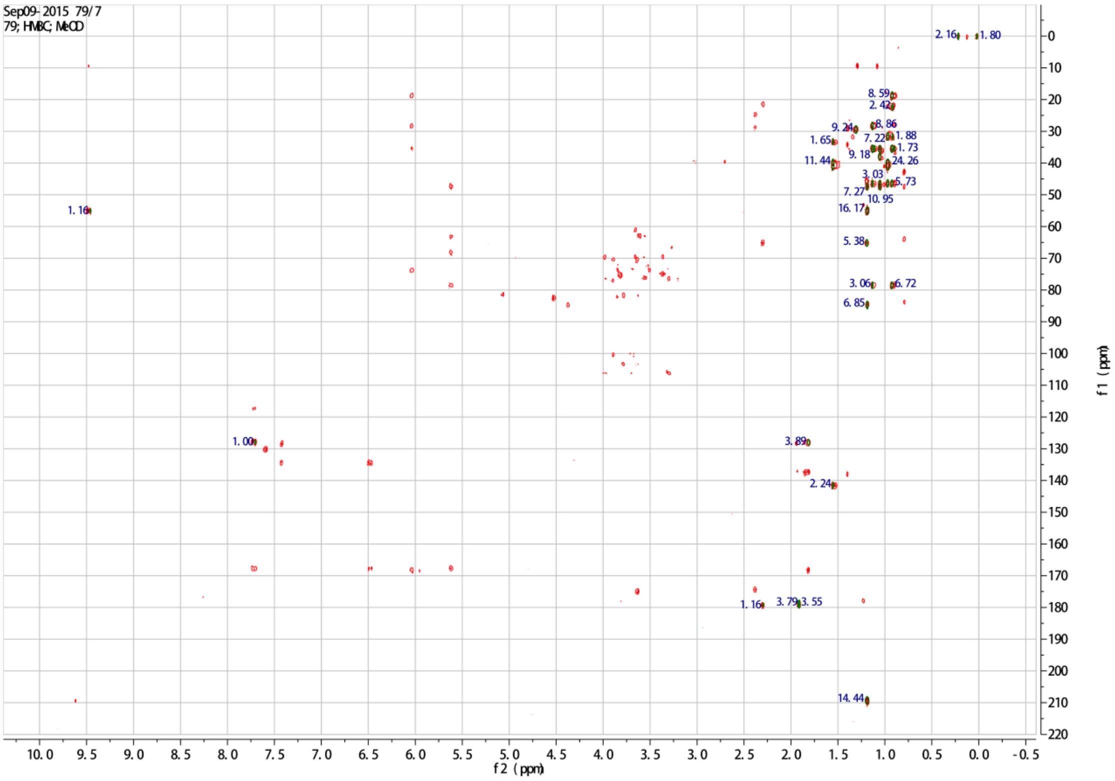

Supplement: Supplementary file 1 [file molecules-22-01562-s001.pdf]
